# Supplementary material for: Belgian Culex pipiens pipiens are competent vectors for West Nile virus while Culex modestus are competent vectors for Usutu virus
Source: PLoS Negl Trop Dis. 2023 Sep 20;17(9):e0011649. doi: 10.1371/journal.pntd.0011649 (PMC10545110; doi:10.1371/journal.pntd.0011649)
Supplement: S1 Fig — USUV AF genome copies were determined by qRT-PCR in individual bodies (A) and heads (B). The bars show the median viral genome copies ± interquartile range; the grey dotted lines represent the limit of detection (LOD) of the qRT-PCR assays used. Statistical analysis was performed with the Mann-Whitney U test. (DOCX) [file pntd.0011649.s001.docx]

###
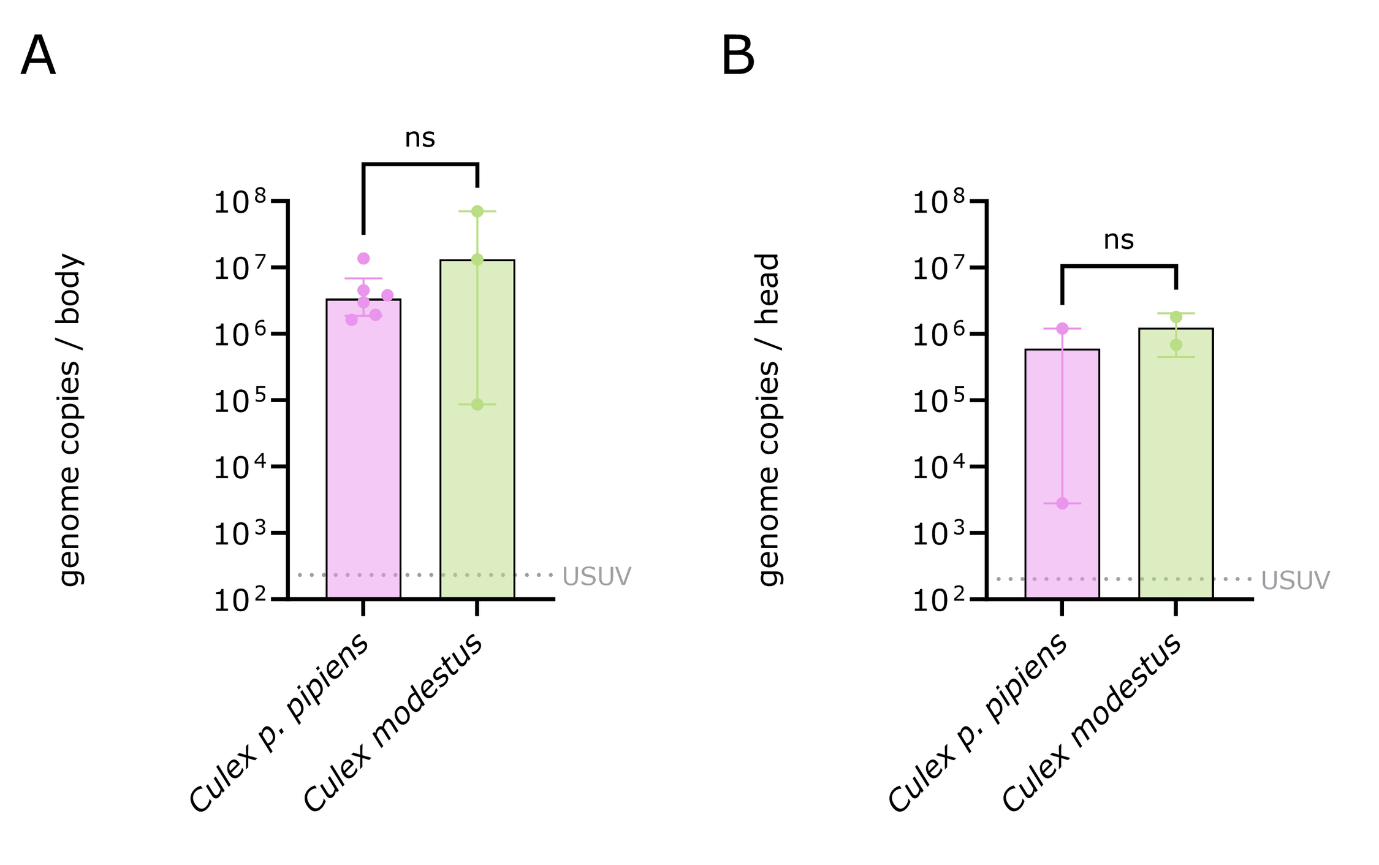
S1 Fig. Comparison of USUV AF genome copies in *Culex pipiens (p.) pipiens* and *Culex modestus*. USUV AF genome copies were determined by qRT-PCR in individual bodies (A) and heads (B). The bars show the median viral genome copies ± interquartile range; the grey dotted lines represent the limit of detection (LOD) of the qRT-PCR assays used. Statistical analysis was performed with the Mann-Whitney *U* test; ns: non-significant.
