# Supplementary material for: Belgian Culex pipiens pipiens are competent vectors for West Nile virus while Culex modestus are competent vectors for Usutu virus
Source: PLoS Negl Trop Dis. 2023 Sep 20;17(9):e0011649. doi: 10.1371/journal.pntd.0011649 (PMC10545110; doi:10.1371/journal.pntd.0011649)
Supplement: S1 Table — (DOCX) [file pntd.0011649.s002.docx]

**S1 Table: Detection of the *Wolbachia wPip* strain in *Wolbachia*-infected *Culex p. pipiens* by virus infection status in the mosquito midgut.**

| **Virus group** | **Midgut infection** | **N mosquitoes** | **% *wPip* positive (n)** |
| --- | --- | --- | --- |
| WNV | Positive | 5 | 100 (5) |
|  | Negative | 5 | 100 (5) |
| USUV EU | Positive | 7 | 100 (7) |
|  | Negative | 7 | 100 (7) |
| USUV AF | Positive | 6 | 100 (6) |
|  | Negative | 6 | 100 (6) |
