# Supplementary material for: Belgian Culex pipiens pipiens are competent vectors for West Nile virus while Culex modestus are competent vectors for Usutu virus
Source: PLoS Negl Trop Dis. 2023 Sep 20;17(9):e0011649. doi: 10.1371/journal.pntd.0011649 (PMC10545110; doi:10.1371/journal.pntd.0011649)
Supplement: S2 Table — The effect of Wolbachia infection on arbovirus infection rate (IR), dissemination rate (DR), and transmission rate (TR) were determined by the Fisher’s exact test. NS: non-significant. (DOCX) [file pntd.0011649.s003.docx]

### S2 Table: Impact of *Wolbachia* infection on virus infection, dissemination, and transmission by species. The effect of *Wolbachia* infection on arbovirus infection rate (IR), dissemination rate (DR), and transmission rate (TR) were determined by the Fisher’s exact test. NS: non-significant.

| **Species** | **Effect on IR, p-value** |  | **Effect on DR, p-value** | **Effect on TR, p-value** |
| --- | --- | --- | --- | --- |
| *Culex p. pipiens* | NS, p=>0.9999 |  | NS, p=>0.9999 | NS, p=>0.9999 |
| *Culex modestus* | NS, p=>0.9999 |  | NS, p=>0.9999 | NS, p=>0.9999 |
